# Diurnal patterns of productivity of arbuscular mycorrhizal fungi revealed with the Soil Ecosystem Observatory

**DOI:** 10.1111/nph.12393

**Published:** 2013-07-12

**Authors:** Rebecca R Hernandez, Michael F Allen

**Affiliations:** 1 Department of Environmental Earth System Science, Stanford UniversityStanford, CA, 94305, USA; 2 Department of Global Ecology, Carnegie Institution for ScienceStanford, CA, 94305, USA; 3 Center for Conservation Biology, University of CaliforniaRiverside, CA, 92521, USA; 4 Department of Biology, University of CaliforniaRiverside, CA, 92521, USA; 5 Department of Plant Pathology and Microbiology, University of CaliforniaRiverside, CA, 92521, USA

**Keywords:** environmental sensors, extra-radical hyphae, plant–fungus, soil organic matter, soil temperature, symbiosis

## Abstract

- Arbuscular mycorrhizal (AM) fungi are the most abundant plant symbiont and a major pathway of carbon sequestration in soils. However, their basic biology, including their activity throughout a 24-h day : night cycle, remains unknown.
- We employed the *in situ* Soil Ecosystem Observatory to quantify the rates of diurnal growth, dieback and net productivity of extra-radical AM fungi. AM fungal hyphae showed significantly different rates of growth and dieback over a period of 24 h and paralleled the circadian-driven photosynthetic oscillations observed in plants.
- The greatest rates (and incidences) of growth and dieback occurred between noon and 18:00 h. Growth and dieback events often occurred simultaneously and were tightly coupled with soil temperature and moisture, suggesting a rapid acclimation of the external phase of AM fungi to the immediate environment.
- Changes in the environmental conditions and variability of the mycorrhizosphere may alter the diurnal patterns of productivity of AM fungi, thereby modifying soil carbon sequestration, nutrient cycling and host plant success.

## Introduction

Of broad interest to scientists are Earth system processes that are modulated by the 24-h day : night cycle (Kayanne *et al*., 1995; Canadell *et al*., 2000; Mears & Wentz, 2005), especially diurnal patterns related to plants (McClung, 2006). For example, the opening of plant stomata by day and their closure at night – that is, in general, for C_3_ and C_4_ pathways – ultimately drives carbon (C) fluxes at ecosystem and global scales (Hetherington & Woodward, 2003). Arbuscular mycorrhizal (AM) fungi that exist symbiotically with the roots of plants also play a critical role in the C cycle, by increasing nutrient and water uptake, which facilitates plant growth, and by providing a C sink in the form of respired C, hyphal mass and glycoproteins, such as glomalin. Extra-radical hyphae, observed in AM fungi (Glomeromycota), grow from the root into the soil in lengths over 100 m cm^−3^ soil (Miller *et al*., 1995), assimilating C received directly from the host plant in exchange for nutrients. The hyphae of AM fungi respire an estimated 3% of fixed C (Paul & Kucey, 1981) and turnover C during the part of the day when host plants are most photosynthetically active (Staddon *et al*., 2003). However, we lack a basic biological understanding of the timing, magnitude and controls of productivity of AM fungi in this external phase at the daily time scale.

Field-based estimates of the productivity of AM fungi have relied on the removal of soil cores for the estimation of the length, diameter and total volume of hyphae (Allen & MacMahon, 1985; Allen & Allen, 1986; Miller & Jastrow 1990; Miller *et al*., 1995). This destructive method precludes the opportunity to measure changes in the hyphal length of AM fungi – that is, growth and dieback – over time. Today, destructive (e.g. soil coring, in-growth mesh bags) and other static ‘snapshot’ methods are still employed for the measurement of various dynamics of productivity of mycorrhizal fungi (i.e. net growth of AM fungal hyphae) and turnover (Miller *et al*., 1995). Using these methods, spatial and temporal variation cannot be separated. Individual soil cores cannot be replicated in time, as the sampled material is destroyed and the change between adjacent points over distances of a few millimeters can be as large as between sample periods (Allen & MacMahon, 1985; Belnap *et al*., 2003). Consequently, the study of hyphal productivity of AM fungi is hampered by logistical problems, including the difficulty in observing microscopic hyphae without concomitantly altering their function, the inability to generate replication in space from which a soil sample is removed and confounding literature from laboratory and glasshouse experiments which may not represent the conditions in natural systems (Allen *et al*., 2007; Vargas & Allen, 2008a,b). For example, Wang *et al*. (1989) found that C was allocated to AM fungal hyphae within 24 h of labeling in a growth chamber experiment, but these rates are unknown in the soil ecosystem.

Treseder *et al*. (2010) utilized a Bartz minirhizotron zoomed in at ×100 to describe the dynamics of single large AM fungal hyphae *in situ* over a season, but the hand imaging and analyses are slow and tedious processes to undertake extensive analyses. Advances in soil imaging automation, environmental sensor technology and digital image processing, together, have conferred the opportunity to observe extra-radical fungal hyphae directly, at unprecedented temporal scales, and in concert with the measurements of variables that characterize the mycelium environment (Allen *et al*., 2007; Rundel *et al*., 2009; Iversen *et al*., 2011).

**Figure 1:**
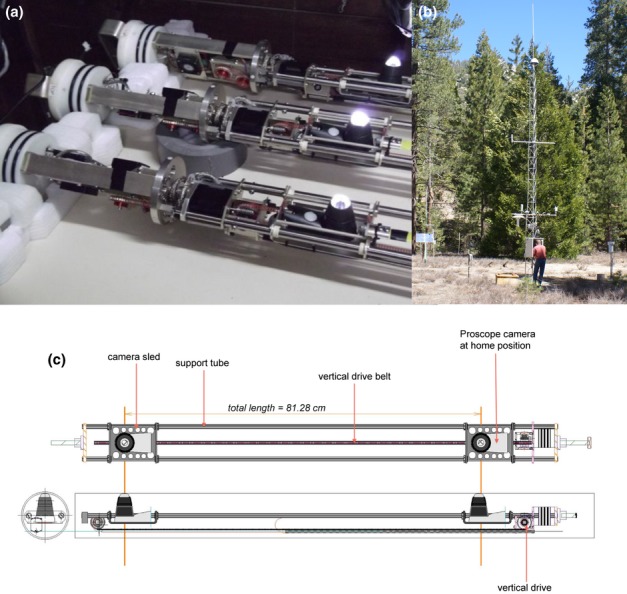
(a) Three individual Soil Ecosystem Observatories (SEOs) without their exterior tubes (note: one single SEO was employed in this study). The Proscope camera was equipped with a 2-megapixel (1.92 million effective pixels) color sensor with a precision of 0.1 mm, accuracy of 0.3 mm and ×100 magnification. The wavelength of the light source was 322 nm. The total length of the SEO is 156.87 cm (107.95 mm in diameter). The maximum imaging area for the entire tube is 320 × 700 mm^2^ with a maximum number of 32 928 images (3.01 × 2.26 mm^2^ each). The SEO has an operating range of −12 to 45°C. (b) Embedded above- and belowground soil sensor network (University of California James San Jacinto Mountains Reserve, Idyllwild, CA, USA) showing the installed SEO (labeled ‘AMR Unit’ in photograph), Campbell CR1000 data logger and Campbell Li-Cor Quantum Sensor. (c) Drawing of the SEO prototype showing the Proscope camera at home position and fastened to the robotic sled.

Like plants, AM fungi may express diurnal-scale patterns of growth, dieback and net productivity, in accordance with endogenous circadian systems or environmental stimuli (Heinemeyer *et al*., 2006); however, to date, no study has tested this directly. Although not suggestive of a diurnal periodicity *per se*, Johnson *et al*. (2002) found that the *in situ* flux of pulse-derived ^13^C peaked in its release as ^13^CO_2_ in AM fungi-colonized soil cores in the first 0–6 h, revealing a rapid allocation of host plant C to AM hyphae. Previous field-based studies on AM rhizomorphs – that is, distinct, large cords comprising several hyphal strands – using *in situ* manual minirhizotrons (Fig.1), showed rapid diurnal changes in rhizomorph length, with growth up to 105.4 cm m^−2^ d^−1^ (Heinemeyer *et al*., 2006; Vargas & Allen, 2008a,b; Hasselquist *et al*., 2010). Given that diel changes in rhizomorphs were dynamic over a 24-h interval, this suggests that individual fungal hyphae may respond similarly. Together, these results suggest that the productivity of mycorrhizal fungi has yet to be quantified at the time scale in which it operates and, if so, any patterns of growth and dieback over a 24-h day : night cycle remain undiscovered.

In addition, environmental stimuli can entrain or reset circadian oscillators, making even circadian rhythms responsive to short-lived external cues, such as light and temperature (Liu & Bell-Pederson, 2006). If the productivity of AM fungal hyphae varies at the diurnal scale, we have increasing evidence that productivity is not only a response to photosynthesis in the host plant (e.g. light stimuli), but also a direct response to changes in the mycorrhizosphere (the zone in which AM hypha–soil interactions occur). For example, Vargas & Allen (2008b) found that rhizomorph growth was positively coupled with soil temperature and precipitation events. In a controlled glasshouse experiment, Heinemeyer *et al*. (2006) found that extra-radical AM fungi increased in length as a result of an increase in temperature. Indeed, glasshouse experiments have shown that AM fungal hyphae are directly modulated by temperature (Heinemeyer *et al*., 2006), but experiments utilizing natural environmental variation are needed to better understand and confirm these relationships.

In this study, our goal was to employ the Soil Ecosystem Observatory as a new method to quantify the diurnal growth, dieback and productivity of extra-radical AM mycorrhizal fungi – the normal condition for *c*. 80% of Earth's terrestrial plant species and most economic crops (Smith & Read, 2008). For this study, we set the temporal resolution of the Soil Ecosystem Observatory to 6-h observations of the soil to discern changes in the dynamics of AM hyphae occurring within a single day. In addition, we tested the hypothesis that AM fungal extra-radical hyphae exhibit differential rates of growth (i.e. elongation) and dieback (i.e. disappearance) throughout a day : night cycle; specifically, that certain 6-h intervals (e.g. 12:00 to 18:00 h, when plant photosynthetic activity is greatest) may show higher rates of growth and dieback than other 6-h intervals within a 24-h time period; furthermore, we examined whether growth and dieback events of AM fungal hyphae are correlated with abiotic variables in the mycorrhizosphere (i.e. soil temperature (*T*_s_), soil moisture (SWS)). Lastly, we were interested in determining whether the growth and dieback of AM fungal hyphae can occur simultaneously and at similar rates, or whether such events are mutually exclusive.

## Materials and Methods

### Study site

This study was conducted at the James San Jacinto Mountains Reserve (http://www.jamesrserve.edu), part of the University of California Natural Reserve System, located at an elevation of 1640 m in the San Jacinto Mountains (Riverside County, CA, USA; 33°48′30″N, 116°46′40″W). Originally, the James Reserve operated as the Terrestrial Ecology Observing System field site, designed for the study of the spatial and temporal dynamics of ecosystem processes using sensor-based technology for the Center for Embedded Networked Sensing (http://research.cens.ucla.edu/; 20).

Surrounded by the San Bernardino National Forest, the James Reserve supports 12 ha of semiarid mixed conifer and oak (*Quercus* sp.) forests. Here, the mean annual precipitation is 507 mm, occurring primarily between November and April, and the mean air temperature is 10.3°C (Vargas & Allen, 2008a). Derived from granitic bedrock, soils at the James Reserve are Entisols, loamy-sand textured (83% sand, 10% silt and 7% clay) and have a bulk density of 1.2 g cm^−3^ (Vargas & Allen, 2008b). Several studies on the physical properties of soils in and surrounding the James Reserve have been published (Hanawalt & Whittaker, 1976; Graham *et al*., 1997; Frazier & Graham, 2000). Previous soil observation-based studies conducted at this site used manual minirhizotrons at ×10 magnification to identify relationships among soil abiotic properties, fine roots and rhizomorphs, at image sampling frequencies of 5 month^−1^ (Vargas & Allen, 2008a,b) and 10 month^−1^ (fine roots only; Kitajima *et al*., 2010). An additional study characterized some daily responses of fine roots and rhizomorphs using observation campaigns (Hasselquist *et al*., 2010).

### The Soil Ecosystem Observatory

We designed and engineered the Soil Ecosystem Observatory (Rhizosystems, LLC, Riverside, CA, USA; Fig.1a,c), a robotic and fully programmable belowground imaging sensor. The Soil Ecosystem Observatory functions in a manner similar to technologies employed for satellite and airborne remote sensing, but, instead, it captures changes in space and time at scales appropriate for soil processes (e.g. cm, μm, h, d). The entire device is 157 cm in length and 108 mm in diameter, with a wide operating range of −12 to 45°C. No measurable changes in soil temperature near the surface of the tube vs 10 cm away were observed (M. F. Allen, unpublished). A robotic sled transports a camera (RGB sensor, 2 megapixels) and light source (wavelength, 322 nm) along the surface of a see-through cylindrical casing, capturing a single tile or image – each 3.01 mm (width) × 2.26 mm (height) – of the soil ecosystem; one image every 2.75 s. At this rate, the observatory can capture over 20 000 high-resolution images every 20 h of operation. The Soil Ecosystem Observatory's total observable area is 2240 cm^2^ and can include up to 32 928 individual images, where each image is captured at a unique point in time and space. The precision and accuracy of the sled-mounted camera are 0.1 and 0.3 mm, respectively. Being fully automated, the Soil Ecosystem Observatory can be programmed remotely to observe and record features in the soil at any spatiotemporal scale of interest.

The Soil Ecosystem Observatory is physically coupled with three belowground sensor nodes, each measuring soil temperature (*T*_s_, HOBO® Weather Station 12-bit Temperature Smart Sensor; Onset, Cape Cod, MA, USA), soil water content (SWC; HOBO*®* Weather Station Soil Moisture Smart Sensor) and CO_2_ concentration (Vaisala®, GMP222; Vaisala, San Jose, CA, USA). The nodes were buried at three different depths (i.e. 2, 8 and 16 cm) alongside the imaging sensor. We used a data logger to program the abiotic sensor network to collect and store data every 5 min (Campbell Scientific, Logan, UT, USA). An LI190SB-L Li-Cor Quantum Sensor (Campbell Scientific) was mounted *c*. 3.3 m above the Soil Ecosystem Observatory and connected to a CR1000 Data Logger (Campbell Scientific). The Quantum Sensor measured the photosynthetic photon flux density (PPFD, μmol m^−2^ s^−1^) in the 400–700-nm waveband every 10 min.

We installed a single Ecosystem Observatory in July 2008 at *c*. 40° declination in a meadow microsite (Fig.1b), > 20 m from the nearest ectomycorrhizal tree, where few if any ectomycorrhizal tips and fungi were observed. Dominant vascular plants included *Artemisia dracunculus* (tarragon), *Eriogonum wrightii* (buckwheat), *Pteridium aquilinum* (bracken fern) and *Bromus tectorum* (cheat grass). Both the molecular sequencing of root tips and direct identification of spores showed the AM fungi at the microsite to be *Glomus* spp.

### Soil Ecosystem Observatory raw data acquisition

In natural soils, AM fungal hyphae are morphologically and architecturally distinct; they are typically thread-like, between 4 and 12 μm in diameter and dichotomously branched (e.g. Allen & MacMahon, 1985; Miller *et al*., 1995; Klironomos *et al*., 1999; Rillig *et al*., 2002; Johnson *et al*., 2003; Treseder *et al*., 2010; Liu *et al*., 2012; Maherali & Klironomos, 2012). We used the context (extension from the root elongation zone), architecture and morphology to resolve them and distinguish them from fine roots and other organisms within individual images captured using the Soil Ecosystem Observatory when set at ×100 magnification (Supporting Information Fig. S1). For this study's purpose, we chose to analyze data from the Soil Ecosystem Observatory collected during 30 d of May in both 2009 and 2010. In this ecosystem, May is a month characterized by dynamic changes in hyphal growth and dieback rates, as it is the end of the growing season.

We randomly selected 20 areas or ‘plots’ (3.01 mm × 2.26 mm) within the Soil Ecosystem Observatory's observable area. Plots without hyphae were excluded and new plots were randomly selected until all 20 plots contained at least one AM fungal hyphal branch. The *x*,*y*,*z* coordinates of each plot were used to determine depth (*z*) below the soil surface employing the depth.exe program (‘depth.exe’; Windows application available from the authors on request). The average depths for images in 2009 and 2010 were 26.88 and 33.50 cm, respectively. Next, all images associated with each plot were queried from every scan conducted from 1 to 31 May. When compiled together, each plot included a time series of *c*. 80 images at a temporal resolution of *c*. three images per day captured at regular intervals within a 24-h day: 00:00–05:59 (I), 06:00–11:59 h (II), 12:00–17:59 h (III) and 18:00–23:59 h (IV). All steps were repeated to create separate 2009 and 2010 time series datasets. Plots in 2009 were spatially different from those in 2010. In total, there were 40 independent plots.

### Image-based data collection in Rootfly

Rootfly (Clemson University, Clemson, SC, USA) is an ecological software application created for minirhizotron image processing in which ‘digitizing’ is a functionality that allows the user to manually trace over a set of complex linear objects and measure their length in a graphic user interface. In this study, all 40 time series were uploaded to Rootfly and all time series were compiled in chronological order (Fig. S2). A border, delineating the boundary of the plot, was digitized in the most recent image (i.e. 31 May) to account for any minor misalignments in the image position resulting from the camera sled when it moves and stops along the tube surface. Engineering upgrades in newer models of the Soil Ecosystem Observatory have since eliminated this shuttling effect. To maintain plot area consistency across the time series, changes in hyphal length outside the plot boundary were not digitized during analyses.

To measure diurnal patterns of hyphal growth and dieback (and, from these, to calculate the rates of productivity), all hyphal branches in the most recent image (e.g. 31 May, 18:00 h) were digitized (manually in Rootfly), assigned a number, and the length of the digitized line as quantified by Rootfly was recorded (Fig. S3). Each hyphal branch had its own unique shape, including curved and angled segments. In Rootfly, every manually digitized segment of a line is inherently straight – a property of the software – and therefore, if a linear object is curved, the user must create several breaks (using mouse clicks), such that a curved line is formed from the creation of numerous, smaller straight segments. Careful attention was paid to duplicate the curves and bends of each segment of the hyphal branch, such that a single branch could easily be comprised of > 50 segments. Images with > 15 branches of hyphae were common and each image could take up to 4 h to digitize. When all branches in a single plot had been numbered and digitized, the digitized lines were copied to the subsequent image (e.g. 31 May, 12:00 h). In the new image, any changes in length (i.e. growth or dieback) and new hyphal branches were edited or digitized, respectively, and their new length measurement values were recorded. When AM fungal hyphae disappeared from an image, we categorized this as mortality. These steps were repeated until the entire set of images in the time series of a single plot had been completed.

For each time series, total hyphal length was calculated by summing the length of all hyphal branches after each scan. The difference in time between subsequent scans was calculated (generated from the Soil Ecosystem Observatory user interface web-based software) to determine the growth and dieback rates (μm mm^−3^ h^−1^). For the majority of the campaign, plots maintained an image sampling frequency of *c*. 6 h. Any changes in AM fungal hyphae observed and recorded from image sampling frequencies of < 15 min or > 6 h between subsequent images were excluded from the analyses, as these could not be associated with the time intervals of interest. In addition, we measured changes in the length of AM fungal hyphae as mm h^−1^ and then converted this into a volumetric value applicable to soil volume (i.e. μm mm^−3^ soil h^−1^) under the assumption that each plot has the dimensions 3.01 mm (length) × 2.26 mm (width) × 0.125 mm (depth) after Merrill & Upchurch (1994) and Ruess *et al*. (2003). Steps were completed for all 40 time series in the 2009 and 2010 campaigns, and there were > 1600 images analyzed in total.

### Statistical analysis

To test our hypothesis that the dynamics of AM fungal hyphae varied at the diurnal time scale, we determined the mean rate of (1) relative growth (μm mm^−3^ h^−1^) and (2) relative dieback (μm mm^−3^ h^−1^) of AM fungal hyphae using plot as the experimental sample unit (*n *=* *40). We also used the nonparametric Kruskal–Wallis chi-squared test to assess differences in ranks, where the null hypothesis is that the median rates of growth (or dieback) are equal across the four 6-h diurnal intervals using R (R: a language and environment for statistical computing 2.11.1). In this analysis, we combined the 2009 and 2010 datasets.

To determine whether the mycorrhizosphere conditions at the study site were similar in 2009 and 2010, differences in all abiotic soil parameters (i.e. at 16 cm depth) between May 2009 and May 2010 were evaluated using Student's paired *t*-test in R. Specifically, we took the mean hourly difference in *T*_s_ and SWC from 1 to 31 May and compared 2009 vs 2010. Although comparing between these years is pseudoreplication, it does not preclude parametric statistical comparison (Hurlbert, 1984).

Each plot varied in the number of events (e.g. new branch formation, reduction in a branch) during a specific interval of time. To account for this, weights were assigned to the mean of each plot where, for example, a mean calculated in a plot based on 10 events was given a higher weight than a mean for a plot based on two events during a single 6-h interval. These weighted plot means were subsequently used to calculate the overall relative mean rate (e.g. growth or dieback) of each 6-h time interval. We used the term ‘relative’ to emphasize that we were calculating the *relative* and not *absolute* differences in the dynamics of AM fungal hyphae across the four 6-h time intervals, as it was not logistically feasible to analyze every plot in the Soil Ecosystem Observatory. Logistical constraints included: (1) the need to restrict the overall spatial extent of the Soil Ecosystem Observatory to achieve its maximum temporal resolution of 6 h; and (2) the human hours required for the manual digitization of the images. Lastly, we calculated 95% confidence intervals for the growth and dieback rates of AM fungal hyphae (for each 6-h interval) using nonparametric bootstrapping in R. In addition, we calculated the rate of net productivity of AM fungal hyphae by summing the rates of growth and dieback. These statistics were repeated for individual campaigns by year (2009 and 2010). Lastly, we evaluated whether the frequencies (i.e. counts) of growth and dieback events were significantly different across the four 6-h day : night intervals (using a chi-squared test) to test whether we would expect to see significantly more growth or dieback activity during a specific interval and, importantly, independent of the magnitude of change.

To determine the mean total relative hyphal length (mm mm^3^ soil), we first summed the length of all hyphal branches in each plot after each scan. Here, we used the term ‘relative’ to emphasize that we were calculating the *relative* total length based on the randomly selected plots and not the *absolute* total length of all plots visible in the Automated Soil Observatory. Next, we calculated the mean total length based on all plots for each day. Standard error (± 1) was calculated for each day. Soil temperature (*T*_s_) and SWC at 16 cm depth were averaged over the 24-h interval, and ± 1SD was calculated over that time interval. Steps were repeated for the 2009 and 2010 campaigns.

To compare relationships between soil abiotic environmental conditions and the dynamics of AM fungal hyphae, *T*_s_ and SWC (16 cm depth) were averaged across each 6-h diurnal time interval for each day. Each hyphal growth or dieback event and its rate were matched, at the same day and time interval, with its respective environmental value. As datasets were relatively large, we developed a program (i.e. R: a language and environment for statistical computing 2.11.1) to automate this step. Subsequently, datasets from 2009 and 2010 were combined. First, we used these data to describe relationships among hyphal growth, hyphal mortality, soil temperature and SWC by calculated weighted regression models (linear, second degree, third degree). Regression models were weighted on the basis of the number of hyphal events and in the same manner as described above. Model I had the form: 

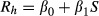
Eqn 1

Model II had the form: 

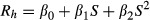
Eqn 2

Model III had the form: 

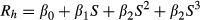
Eqn 3

The best fit parameters were calculated for each model, together with the squared coefficient of regression (*r*^2^), the *F* statistic, the residual standard error (SE) and the *P* value. We calculated the Akaike information criterion (AIC) from the equation: 

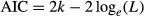
Eqn 4

AIC is derived from the number of parameters (*k*) in the statistical model, where *L* is the maximized value of the likelihood function for the estimated model, and the lowest AIC value denotes the preferred model. In this study, AIC was used as a penalized likelihood criterion for comparison between models (Burnham & Anderson, 2002). Lastly, we archived a selection of AM fungi and sensor data from this study in the DataONE member node, the Knowledge Network for Biocomplexity (http://knb.ecoinformatics.org; see Hernandez & Allen, 2013).

## Results

### Diurnal growth and dieback of AM fungal hyphae

**Figure 2:**
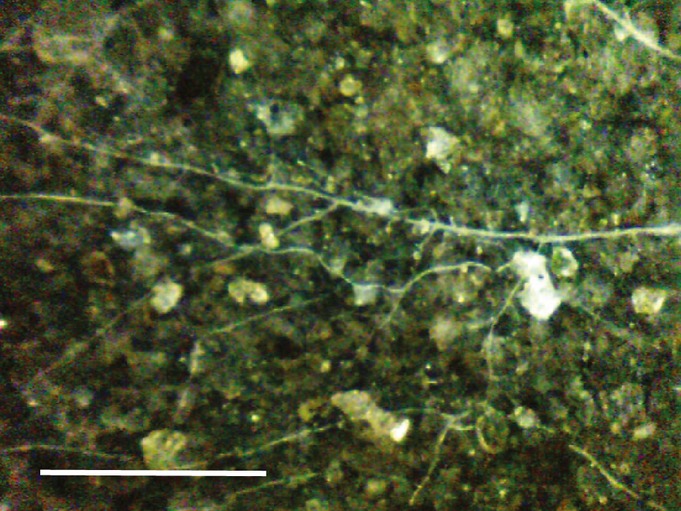
Belowground image (3.01 × 2.26 mm^2^) of arbuscular mycorrhizal fungal hyphae captured with the Soil Ecosystem Observatory (SEO). The SEO was installed in a mixed conifer forest at the James San Jacinto Mountains Reserve, which operates as a field site for the Center for Embedded Networked Sensing (Idyllwild, CA, USA). The image was taken at × 100 magnification; bar, 1 mm.

**Figure 3:**
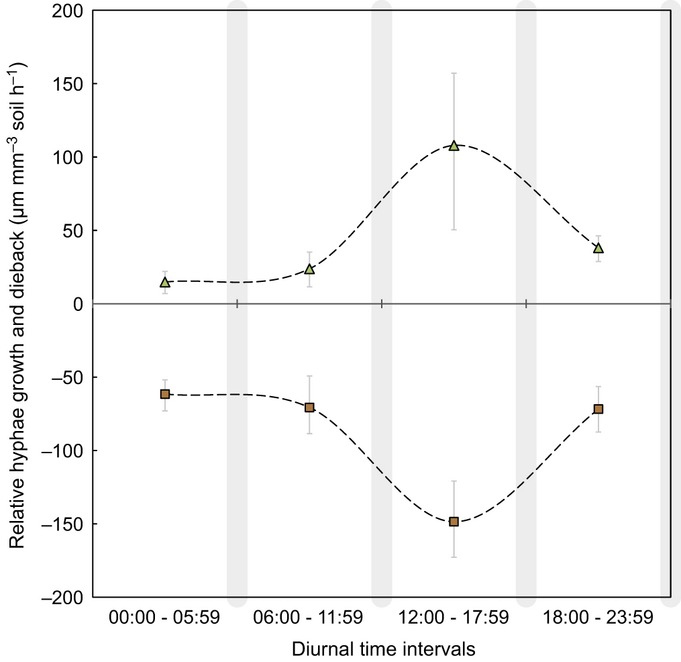
Relative diurnal growth (green triangles; Kruskal–Wallis *χ*^2^ = 24.2814, df =* *3, *P *=* *2.182e-05) and dieback (brown squares; Kruskal–Wallis *χ*^2^ = 63.9063, df* *=* *3, *P *=* *8.596e-14) rates of extra-radical arbuscular mycorrhizal hyphae (μm mm^−3^ soil h^−1^). Data quantified from 40 time series analyses of automated soil observatory images (*n *>* *1600) over a 30-d field campaign in May 2009 and 2010. Error bars are 95% confidence intervals (nonparametric bootstrapping).

We found that the diurnal dynamics of AM fungal hyphae were visible – producing images of *in situ* and extra-radical AM fungal hyphae (Fig.2) – and quantifiable with the Soil Ecosystem Observatory. Using this new technology, we found that the rates of relative growth and dieback of AM fungal hyphae were significantly different across the four 6-h intervals comprising a 24-h day : night cycle (Fig.3; Kruskal–Wallis *χ*^2^* *=* *25.505, df* *=* *3, *P* < 0.0001 (production); Kruskal–Wallis *χ*^2^* *=* *77.5848, df* *=* *3, *P *< 0.0001 (mortality)). Peak growth rates of AM fungal hyphae occurred between 12:00 and 17:59 h, with a mean growth rate of 154 μm mm^−3^ soil h^−1^. This rate of growth was approximately four times greater than the growth observed in other intervals, which ranged from 27 to 47 μm mm^−3^ soil h^−1^. Similar to growth, the relative dieback rates of AM fungal hyphae were greatest between 12:00 and 17:59 h, and slightly greater than growth (170 μm mm^−3^ soil h^−1^). Dieback in nonpeak intervals ranged from 63 to 102 μm mm^−3^ soil h^−1^. Hyphal dieback and growth showed a clear minimum in the morning (i.e. 00:00–06:00 h) with rates of 63 and 27 μm mm^−3^ soil h^−1^, respectively.

**Figure 4:**
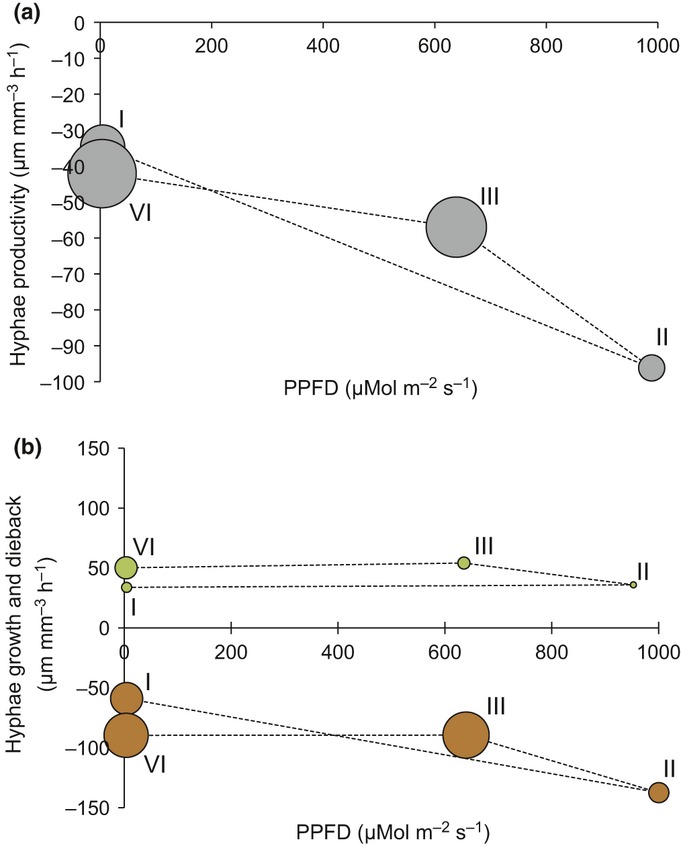
Mean relative diurnal (a) productivity (gray circles; μm mm^−3^ soil h^−1^) and rates of (b) growth (green circles) and dieback (brown circles) as a function of the mean photosynthetic photon flux density (gray circles; μmol m^−2^ soil s^−1^) during four 6-h intervals within a 24-h day: 00:00–05:59 h (I), 06:00–11:59 h (II), 12:00–17:59 h (III) and 18:00–23:59 h (IV). Circle size is proportional and indicative of the number of growth and dieback events (i.e. independent of the rate of change; gray, both growth and dieback events; green, growth events; brown, dieback events) of arbuscular mycorrhizal (AM) fungal hyphae throughout the four day : night intervals.

We also found that the frequency (i.e. counts) of growth and dieback events was significantly different across the four 6-h day : night intervals, independent of the rate of change (Fig.4a; *χ*^2^* *=* *31.803, df* *=* *3, *P *<* *0.0001). The greatest activity – in both directions (i.e. growth and dieback) – was observed in intervals III (12:00–17:59 h) and IV (18:00–23:59 h), whereas only 10.0% of all activity was observed in interval II. Analyzed separately, growth and dieback events showed the same pattern across the 6-h intervals, where the greatest number of events occurred during intervals III and IV, and the least in interval II (Fig.4b; *χ*^2^* *=* *16.694, df* *=* *3, *P *<* *0.0008 and *χ*^2^* *=* *19.679, df* *=* *3, *P *<* *0.0002, respectively).

### Diurnal dynamics of AM fungal hyphae by campaign year

In 2009, the observed mean *T*_s_ for the month of May was 17.5°C (16 cm depth), with a monthly minimum : maximum of 11.5 : 22.4°C. The daily *T*_s_ range – defined as the long-term mean of the daily difference between the maximum and minimum soil temperature – was 5.6°C, lower when compared with the total monthly range of 10.9°C. The observed mean SWC was 0.078 mm^3^ mm^−3^ (16 cm depth), with a monthly minimum : maximum of 0.120 : 0.118 mm^3^ mm^−3^. The daily SWC range was 0.006 mm^3^ mm^−3^, higher when compared with the total monthly range of 0.004 mm^3^ mm^−3^.

In 2010, the observed mean *T*_s_ for the month of May was 13.2°C (16 cm depth), with a monthly minimum : maximum of 7.2 : 18.5°C. The daily *T*_s_ range was 5.1°C, lower when compared with the total monthly range of 11.3°C. The observed mean SWC was 0.187 mm^3^ mm^−3^ (16 cm depth), with a monthly minimum : maximum of 0.166 : 0.227 mm^3^ mm^−3^. The daily SWC range was 0.006 mm^3^ mm^−3^, lower when compared with the total monthly range of 0.061 mm^3^ mm^−3^.

Soil conditions (i.e. *T*_s_, SWC; 16 cm depth) were significantly different between 2009 and 2010 (Fig.3). Diurnal soil temperatures were significantly lower in 2010 than in 2009 (paired Student's *t*-test; *t *=* *34.3548, df* *=* *23, *P* < 0.0001). Furthermore, SWC was significantly greater in 2010 than in 2009 (paired Student's *t*-test; *t *=* *−748.6821, df* *=* *23, *P* < 0.0001).

As a result of the differences observed in *T*_s_ and SWC between 2009 and 2010, we also analyzed the diurnal-scale behavior of AM fungal hyphae by campaign year (Fig. S4). Relative rates in 2010 were lower than those in 2009, paralleling the lower and more variable soil temperature. In 2009, the relative rates of growth (183.4 μm mm^−3^ soil h^−1^) and mortality (–206.4 μm mm^−3^ soil h^−1^) were greatest between 12:00 and 17:59 h (i.e. interval III). In addition, hyphal growth in 2009 was significantly different across time intervals (Kruskal–Wallis *χ*^2^* *=* *25.505, df* *=* *3, *P *=* *0.211e-05). Specifically, the growth in time interval III was *c*. five, three and four times greater than the growth in intervals I, II and IV, respectively. Like growth, hyphal dieback rates were also significantly different across time intervals (Kruskal–Wallis *χ*^2^ = 77.5848, df = 3, *P* = 2.20e-16). Lastly, growth (33.9 μm mm^−3^ soil h^−1^) and dieback (−57.8 μm mm^−3^ soil h^−1^) were lowest in interval I.

In 2010, the diurnal patterns of AM fungal hyphae differed notably from the patterns observed in 2009. For example, the relative growth was greatest from 18:00 h to 23:59 h and was nearly an order of magnitude less (i.e. 23.1 μm mm^−3^ soil h^−1^) than the greatest growth mean observed in 2009. Growth did not differ significantly across diurnal time intervals (Kruskal–Wallis *χ*^2^* *=* *0.4535, df* *=* *3, *P *=* *0.929), whereas dieback rates did (Kruskal–Wallis *χ*^2^ = 10.741, df =* *3, *P *=* *0.01321). Similar to 2009, dieback was greatest between 12:00 h and 17:59 h at −75.4 μm mm^−3^ soil h^−1^, yet this, too, was much lower than the dieback rates observed in interval III in 2009.

### Net productivity of AM fungal hyphae

**Figure 5:**
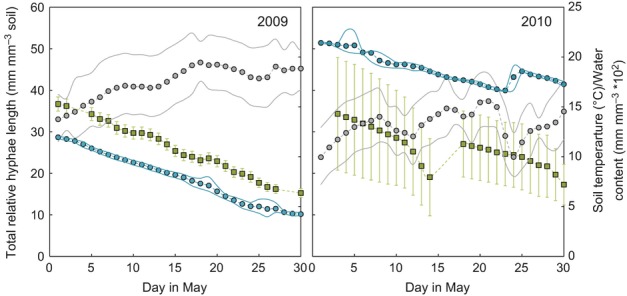
Mean total relative hyphal length (gray squares; mm mm^−3^ soil) of arbuscular mycorrhizal fungi during the month of May 2009 and 2010. Error bars are ± 1SE. Soil temperature (gray circles; °C) and soil water content (blue circles; mm mm^−3^ × 10^2^) at 16 cm depth on secondary *y*-axis with solid lines showing ± 1SD.

Overall, the rates of dieback of AM fungal hyphae during both campaigns were approximately two-fold greater than hyphal growth at *c*. −104 vs 68 μm mm^−3^ soil h^−1^, respectively, averaged across all intervals, for a net production rate of −36 μm mm^−3^ soil h^−1^ (Fig.5). This disparity resulted in a net reduction in length. Specifically, the total length was reduced by over one-half throughout both the 2009 and 2010 May campaigns (e.g. from *c*. 36 to 18 mm^3^ mm^−3^ soil). A net reduction in length for the month of May parallels our findings of net negative AM fungal hyphal rates calculated at the diurnal scale.

### Dynamics of AM fungal hyphae correlate with abiotic soil properties

Next, we elucidated the relationships among the rates of growth and dieback of AM fungal hyphae and the productivity quantified from the Soil Ecosystem Observatory and soil temperature, SWC and PPFD derived from sensor observations. Of several models explaining the relationship between the growth of AM fungal hyphae and temperature, the best model was a second-degree, nonlinear function (multiple *R*^2^* *=* *0.4138, SE* *=* *11.9, *P *=* *0.008178; Table1). In this model, growth is minimal (i.e. below 20 μm mm^−3^ soil h^−1^) when temperatures are in the range 13–15°C, but increases quadratically as temperatures increase/decrease beyond this range. Dieback was best explained with a linear model (multiple *R*^2^* *=* *0.3707, SE* *=* *10.68, *P *=* *0.003398) in which length decreased by 5 μm mm^−3^ soil h^−1^ for every 1°C increase in temperature. The best relationship between hyphal growth and SWC was also linear (multiple *R*^2^* *=* *0.4336, SE* *=* *12.48, *P *=* *0.002963), with hyphal growth decreasing by 14 μm mm^−3^ soil h^−1^ for every 0.05 mm^3^ mm^−3^ increase in SWC. No significant relationship was identified between hyphal dieback and SWC.

**Table 1:** Results of weighted regression models relating arbuscular mycorrhizal fungal hyphae dynamics (i.e. growth, dieback; *R*_h_) to soil abiotic variables (S): soil temperature (*T*_s_) and soil water content (SWC)

Variables	Model	*β*_0_	*β*_1_	*β*_2_	*β*_3_	*r* ^2^	*F*-statistic	Residual SE	*P*-value	AIC
Growth										
*T*_s_	I	−41.169	4.522	–	–	0.2765	7.261	12.87	0.01435*	201
	II	213.205	−27.840	0.996	–	0.4138	6.352	11.9	0.008178**	198
	III	−29.153	21.791	−2.290	0.070	0.4225	4.146	12.16	0.02239*	200
SWC	I	68.06	−274.95	–	–	0.4336	12.25	12.48	0.002963**	161
	II	91.96	−721.33	1730.15	–	0.4519	6.184	12.68	0.01100*	163
	III	75.74	−248.71	−2432.80	11176.78	0.4539	3.878	13.1	0.03285*	164
Dieback										
*T*s	I	7.972	−5.061	–	–	0.3707	11.19	10.68	0.003398**	195
	II	−121.402	13.128	−0.611	–	0.5068	9.246	12.5	0.001728**	200
	III	690.589	−153.153	10.395	−0.236	0.5818	7.885	11.84	0.001640**	198
SWC	I	−109.38	246.81	–	–	0.1783	3.69	16.96	0.07168	193
	II	−182.20	1672.18	−5378.90	–	0.2579	2.78	16.62	0.09203	193
	III	−188.2	1758.8	−6112.0	1885.9	0.2579	1.737	17.16	0.2022	195

Model I has the form *R*_h_ = *β*_0_ + *β*_1_(S), model II the form *R*_h_ = *β*_0_ + *β*_1_S + *β*_2_S^2^, and model III the form *R*_h_ = *β*_0_ + *β*_1_S + *β*_2_S^2^ + *β*_2_S^3^. The best fit parameters are reported for each model together with the squared coefficient of regression (*r*^2^), the *F*-statistic, the residual standard error (SE), and the Akaike information criterion (AIC). Asterisks denote significance (^*^, *P* < 0.05; ^*^^*^, *P* < 0.01).

### Diurnal patterns of AM fungal hyphae – a conceptual model

**Figure 6:**
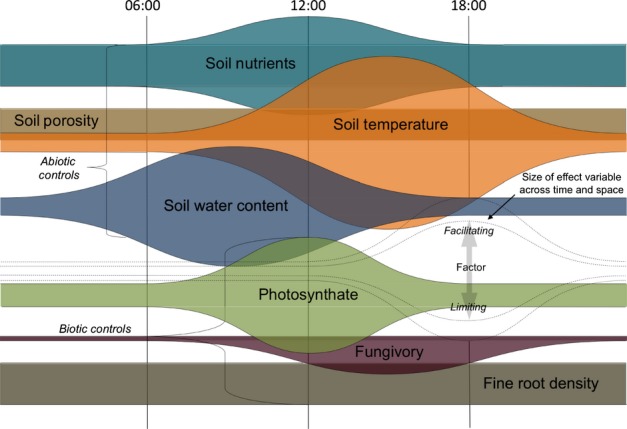
A conceptual model illustrating the properties of the mycelium environment that may exogenously influence (or entrain endogenous circadian oscillators controlling) the productivity of extra-radical arbuscular mycorrhizal (AM) fungal hyphae at the diurnal scale. In this model, factors impacting the productivity of AM fungal hyphae include fungivory, photosynthate input, soil nutrient availability, soil temperature and soil water content. Changes in fine root density and porosity impact AM fungi at coarser periods of time, but are relatively constant over this time scale, and thus are not included as model factors. The size of the bulge of each potential factor corresponds to its impact on the hyphal network. These impacts vary across time and space in the hyphosphere and can be facilitating (i.e. promoting elongation; upward slope) or limiting (i.e. promoting dieback; downward slope).

Lastly, to showcase the rather complex set of factors influencing the sub-daily dynamics of AM fungal hyphae, we characterized the properties of the mycelium environment that may exogenously influence (or entrain endogenous circadian oscillators that control) the observed diurnal temporal variation in the production of AM fungal hyphae. We created a conceptual model, informed from this and previous studies (Liu & Bell-Pederson, 2006; Smith & Read, 2008), to describe these potential diurnal-scale controls of AM fungal hyphae (Fig.6). In this model, factors impacting on the productivity of AM fungal hyphae include fungivory, photosynthate input, soil nutrient availability, *T*_s_ and SWC. Changes in fine root density and porosity impact on AM fungi at coarser periods of time, but are relatively constant over this time scale, and thus are not dynamic model factors. The size of the bulge of each potential factor corresponds to its impact on the hyphal network. These impacts will probably vary across time and space in the mycorrhizosphere, and can be facilitating (i.e. promoting elongation; upward slope) or limiting (i.e. promoting dieback; downward slope).

## Discussion

### Diurnal patterns of AM fungal hyphae in a natural environment

The description of the diurnal patterns of plant physiology and their relationship with environmental factors has been of interest to scientists for over 60 yr (Larcher, 2003). From early on, scientists recognized the challenge and importance of taking plant ecophysiological measurements in natural settings (Tenhunen *et al*., 1987; Mooney & Field, 1989; Mooney, 1991). Such *in situ* measurements of short-term dynamics provided the foundation for the elucidation of not only whole-plant function, but ecosystem level and Earth system processes (Mooney & Field, 1989; Zotz & Winter, 1993; Hetherington & Woodward, 2003; McClung, 2006). In comparison, relatively little progress was made – and still remains the case today – in understanding the diurnal patterns of the plant–fungus symbiosis, an essential relationship if we wish to truly describe plant physiology and function as a ‘whole’. Observations of AM fungi in natural settings are essential because of the inherent complexity of the plant–fungi symbioses (Treseder, 2004) in which fungi exhibit plant-dependent and -independent responses, and such responses both create and are impacted by numerous feedbacks. To this end, understanding the short-term dynamics of AM fungi requires an understanding of plant-dependent and -independent responses simultaneously, together with their associated feedbacks, which can only be accomplished in a natural setting.

Before this study, the timing of the growth of extra-radical hyphae (and their rate of growth) during a 24-h day : night cycle was not well understood. However, several studies made prescient hypotheses alluding to their dynamism based on the rapid allocation of labeled ^11^C to plant roots and mycorrhizal fungi in a phytotron (Wang *et al*., 1989) and ^14^CO_2_ to soil cores colonized by AM (Johnson *et al*., 2002), measurements of rhizomorph growth (Vargas & Allen, 2008a,b; Hasselquist *et al*., 2010), rates of coarse hyphal production (Treseder *et al*., 2010) and standing crop estimates of AM hyphae interpolated from soil core measurements (Miller *et al*., 1995). In this study, we determined that the rates of *in situ* AM fungal productivity in the meadow of a semiarid mixed conifer forest fluctuated throughout a 24-h day : night period. Growth rates and incidences of elongation were maximal from 12:00 to 17:59 h – when elongation often exceeded 150 μm mm^−3^ h^−1^ and soils were at peak temperature. Similar to the circadian and exogenously driven oscillation system that drives the photosynthesis observed in plants, hyphal elongation may be a sinusoidal pattern, peaking in the same 6-h interval when C_3_ and C_4_ plant productivity is also greatest (Larcher, 2003).

Patterns of dieback paralleled growth, with incidences and rates of dieback of AM fungal hyphae being greatest in the interval from 12:00 to 17:59 h. Reductions in external hyphae are important to understand at the diurnal scale because, like growth, hyphal dieback may influence phosphorus inflow to the host plant (Liu *et al*., 2012). However, unlike growth, increased hyphal dieback at diurnal timescales may be indicative of changes in resource allocation from belowground to shoots and leaves (Bloom *et al*., 1985; Johnson *et al*., 2003, 2008) and/or fungivory, and also regulates directly the flow of C, either as throughput or as more stable sources, into the soil. Future studies exploring the diurnal patterns of dieback of AM fungal hyphae should evaluate how such rates and incidences of dieback differ throughout the rhizosphere (e.g. depth, distance from host plant) and across ecosystems.

Our observations suggest the existence of a biotic (e.g. photosynthate availability) and abiotic (e.g. temperature) ‘optimum’ for AM fungal networks, conditions that probably operate synergistically to facilitate hyphal productivity through the soil. In that vein, the term ‘optimal’ may be somewhat misleading. This is because resource limitations (e.g. C, phosphorus, nitrogen), in the host plant or its symbiont, may also drive elongation (Orwin *et al*., 2011). Further underscoring the complexity of the conditions influencing changes within a mycelium network, our study showed that the growth and mortality of AM fungal hyphae occurred throughout the entire 24-h day : night period, even when host plants ceased light-dependent photosynthesis and PPFD was negligible. Consequently, lags and factors external to the plant–fungi symbiosis probably further impact the timing and magnitude of the productivity of AM fungal hyphae.

We anticipated a net reduction in AM fungal standing crop, as May is the end of the growing season in this Mediterranean-type ecosystem (for details, see Vargas & Allen, 2008a). Integrating across all intervals, our study provides an estimate of daily production at 1.6 mm mm^−3^ and mortality at 2.5 mm mm^−3^ in the mycorrhizosphere in May. This equates to a loss of roughly 0.9 mm mm^−3^ mycelium per day throughout our campaigns. As this ratio of growth : dieback will probably change throughout the year and by ecosystem type, future studies should evaluate diurnal patterns of growth and dieback during biologically important times throughout the year and in different ecosystems to better understand the annual rates of productivity.

Of importance for understanding AM fungal turnover, we observed anecdotally that runner hyphae (coarser in diameter) persisted throughout most of both campaigns, whereas hyphae strands thinnest in diameter were most likely to die back. The most dynamic hyphae are part of the absorbing network tips, which have been shown previously to respond to rapid stimuli (Friese & Allen, 1991). These data also confirm the observations of Treseder *et al*. (2010), who noted that the coarse hyphae had a residence time of > 145 d during the summer drought. Our observations also addressed the question of whether hyphal elongation and dieback are mutually exclusive within the mycorrhizosphere. Much like the simultaneous growth and senescence of different leaves on a tree, we found that elongation and dieback can occur concomitantly within AM fungal networks. Consequently, future studies should not only elucidate the factors impacting production, but also tease apart their roles in facilitating or inhibiting elongation and dieback events separately.

### Short-term dynamics of AM fungal hyphae, respiration and abiotic soil conditions

In laboratory and glasshouse experiments, several studies have shown that AM hyphae are modulated directly by abiotic conditions in the soil (Monz *et al*., 1994; Rillig *et al*., 2002; Gavito *et al*., 2003; Alberton *et al*., 2005; Heinemeyer *et al*., 2006, 2007; Vicca *et al*., 2009; Cheng *et al*., 2012). For example, Monz *et al*. (1994) reported that the colonization by AM fungi in two host plants decreased when precipitation increased, and one plant showed reduced colonization in response to increasing temperature – patterns also observed in our study. Because our study conferred direct observations and measurements in a natural habitat, our findings confirm previous hypotheses on the acclimation of extra-radical AM hyphae to temperature.

AM fungal hyphae also appear to respond rapidly, potentially even to hourly changes in response to host and diurnal environmental cycles. Staddon *et al*. (2003) found a turnover of hyphae within 5–6 d based on soil ^14^C studies, showing that these fungi respond markedly at short time scales. At a nearby meadow to the current study, during May of 2006 (phase III of their study), using a Bartz conventional minirhizotron and the networked sensor instrumentation equivalent to that described here, soil respiration responded directly to soil temperature and moisture, showing no hysteresis, at a 24-h time scale (Vargas & Allen, 2008b). Soil respiration was correlated primarily with soil temperature, vapor pressure deficit (VPD) and photosynthetically active radiation (PAR). Putting our observations into these contexts, the growth and respiration of hyphae appear to be tightly coupled to the environmental and plant growth drivers. The primary environmental variable changing within the 24-h cycle is soil temperature, peaking in the afternoon, into the evening. The plant variables are VPD, which drives transpiration, and thus CO_2_ exchange through the stomata, and PAR, which drives CO_2_ fixation. Thus, hyphal production and soil respiration are co-correlated with each other and with critical environmental (temperature) and plant photosynthetic (VPD, PAR) variables that cycle on a diurnal scale.

Hyphal production was more dynamic in 2010 – when PPFD and soil temperatures showed higher variability – than in 2009. It is also clear that AM fungal hyphae are dynamic within a short (24 h) period of time. AM fungi clearly are modulated directly (exogenously; e.g. temperature) and indirectly (e.g. circadian oscillators, assimilation in host plant), as suggested in several studies (Monz *et al*., 1994; Rillig *et al*., 2002; Gavito *et al*., 2003; Alberton *et al*., 2005; Heinemeyer *et al*., 2006, 2007; Cheng *et al*., 2012). Consequently, net growth of AM fungal hyphae may be impacted by short-term weather events (e.g. heat-waves and droughts) and also longer term changes in weather, such as climate change (Rillig, 2004; Govindarajulu *et al*., 2005; Cheng *et al*., 2012).

### Summary

Our study's novel technological approach, the Soil Ecosystem Observatory, revealed that the productivity of AM fungal hyphae and its interaction with the soil ecosystem is more complex than previously thought, requiring a nondestructive and observation-based experimental approach. First, our study showed that AM fungal hyphae are highly dynamic across a diurnal time scale, with maximal rates and incidences of growth and mortality occurring from noon to 17:59 h. Production and mortality rates were tightly coupled with diurnal-scale changes in temperature and hydrology within the mycorrhizosphere. Second, we found that elongation and dieback can occur simultaneously within a single AM fungal network, with the magnitude of each rate determining whether there is a net input to the soil. In addition, our study suggests that variability in soil abiotic factors may increase variability in hyphal production and mortality rates, such as those observed in 2010. Lastly, our study underscores the vast potential of Soil Ecosystem Observatories to elucidate soil microbial function where direct human observation and measurements are otherwise impossible. This is a leap forward in the understanding of mycorrhizal fungal ecology, as past studies have typically measured processes on weekly or monthly time intervals, and have relatively overlooked extra-radical AM mycelium.

## Supporting Information

**Fig. S1***In situ* Soil Ecosystem Observatory images showing morphological differences between fine roots and arbuscular mycorrhizal (AM) fungal hyphae. **Fig. S2** A subset of a time series of Soil Ecosystem Observatory images showing changes in arbuscular mycorrhizal (AM) fungal hyphae in one plot within a single day. **Fig. S3** A Soil Ecosystem Observatory image/plot as digitized in Rootfly. **Fig. S4** Diurnal growth and dieback rates of arbuscular mycorrhizal (AM) fungal hyphae in 2009 and 2010.
