# Supplementary material for: Diurnal patterns of productivity of arbuscular mycorrhizal fungi revealed with the Soil Ecosystem Observatory
Source: New Phytol. 2013 Jul 12;200(2):547–57. doi: 10.1111/nph.12393 (PMC4288973; doi:10.1111/nph.12393)
Supplement: Supplementary file 1 — Fig. S1In situ Soil Ecosystem Observatory images showing morphological differences between fine roots and arbuscular mycorrhizal (AM) fungal hyphae. Fig. S2 A subset of a time series of Soil Ecosystem Observatory images showing changes in arbuscular mycorrhizal (AM) fungal hyphae in one plot within a single day. Fig. S3 A Soil Ecosystem Observatory image/plot as digitized in Rootfly. Fig. S4 Diurnal growth and dieback rates of arbuscular mycorrhizal (AM) fungal hyphae in 2009 and 2010. [file nph0200-0547-sd1.doc]

**Supporting Information Figs S1–S4**

**Fig. S1** *In-situ* Soil Ecosystem Observatory images showing fine roots and AM fungal hyphae.

**Fig. S2** A subset of a time series of AM fungal hyphae images.

**Fig. S3** AM fungi in a Soil Ecosystem Observatory image digitized in Rootfly.

**Fig. S4** Diurnal-scale patterns of AM fungal hyphae productivity by year (2009 and 2010).


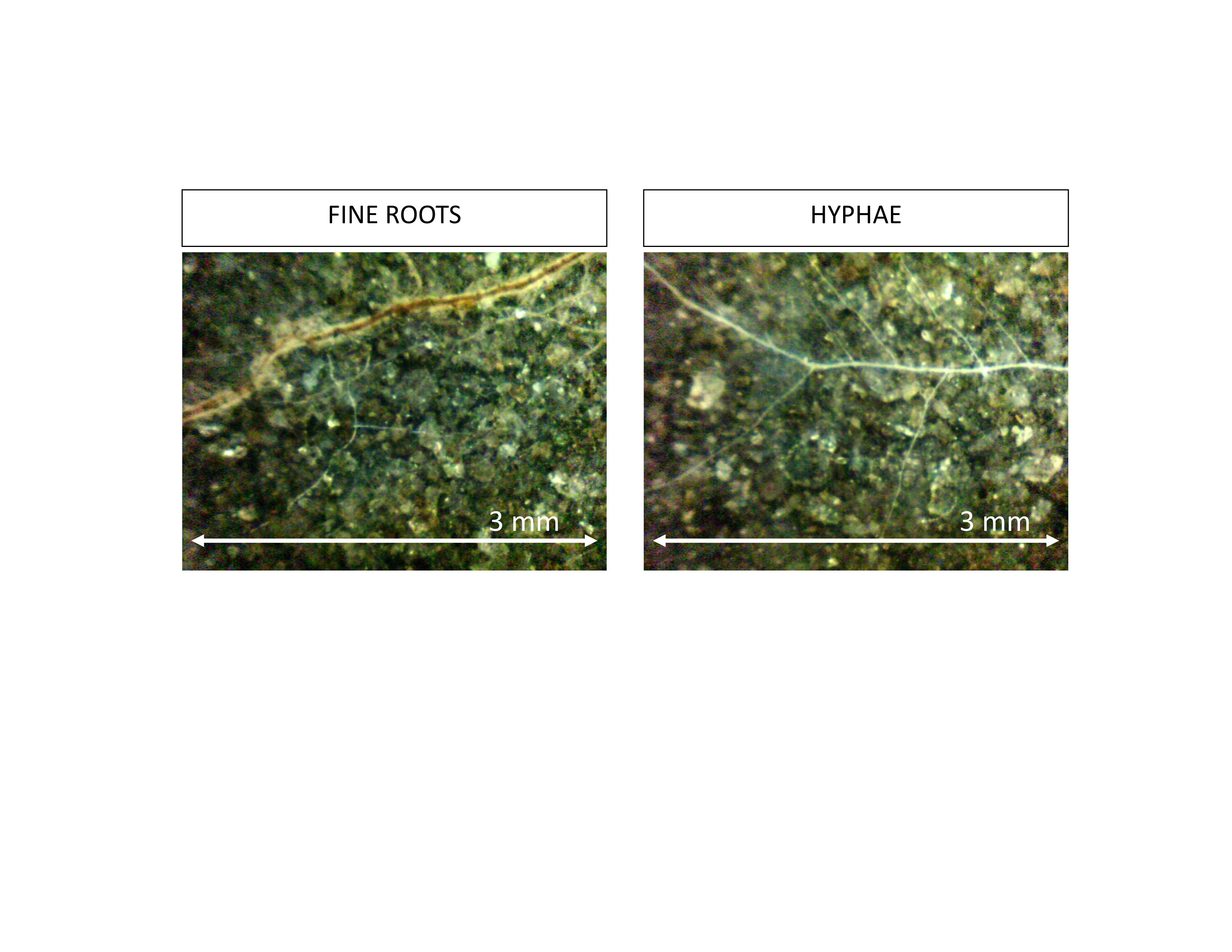


**Fig. S1** *In-situ* Soil Ecosystem Observatory images (×100) showing the morphological differences between fine roots (left) and AM fungal hyphae (right).


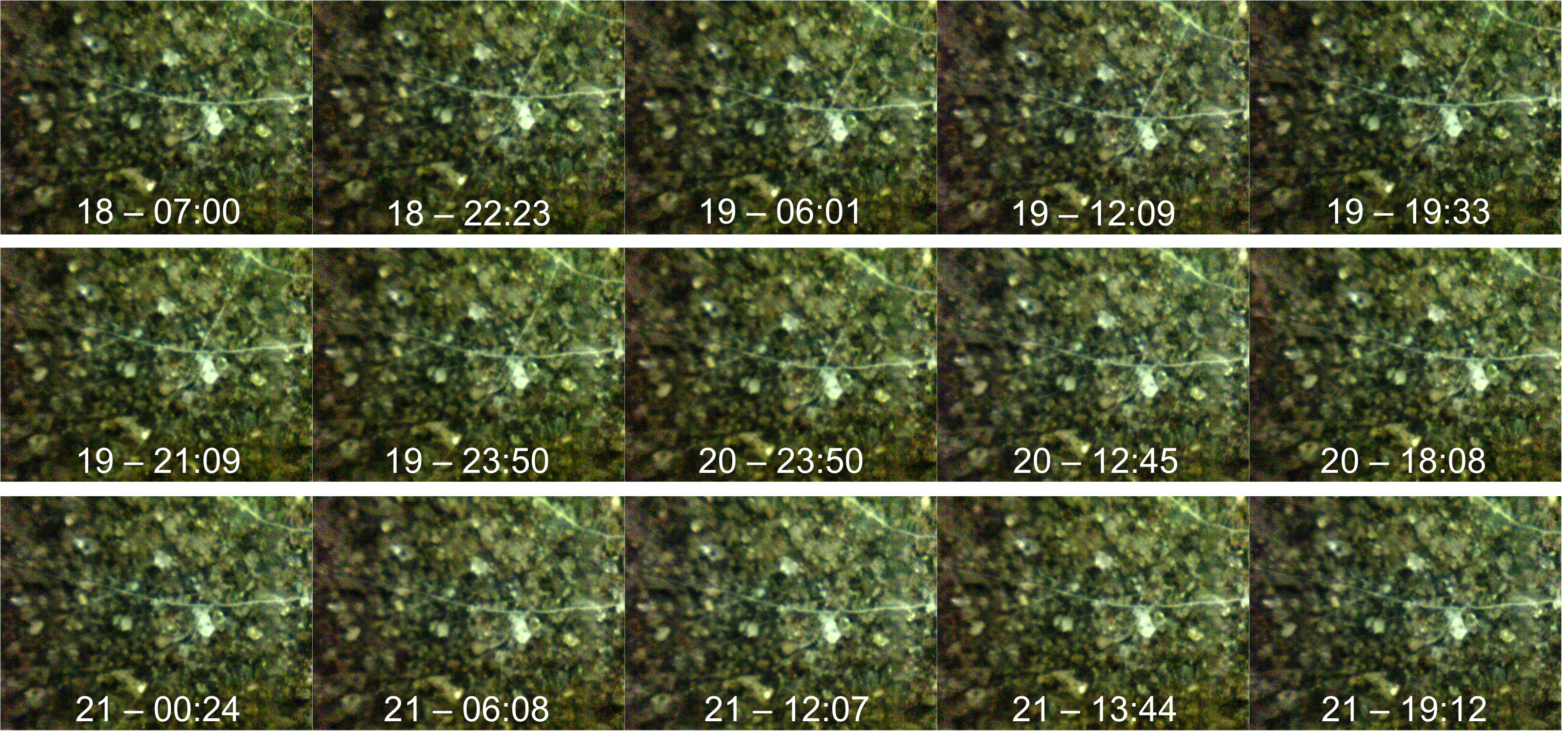


**Fig. S2** A subset of a time series of Soil Ecosystem Observatory images (×100, a single plot) from 18 to 21 May 2010 showing diurnal changes in AM fungi hyphae length in one plot. Annotations are DD – HH:MM.


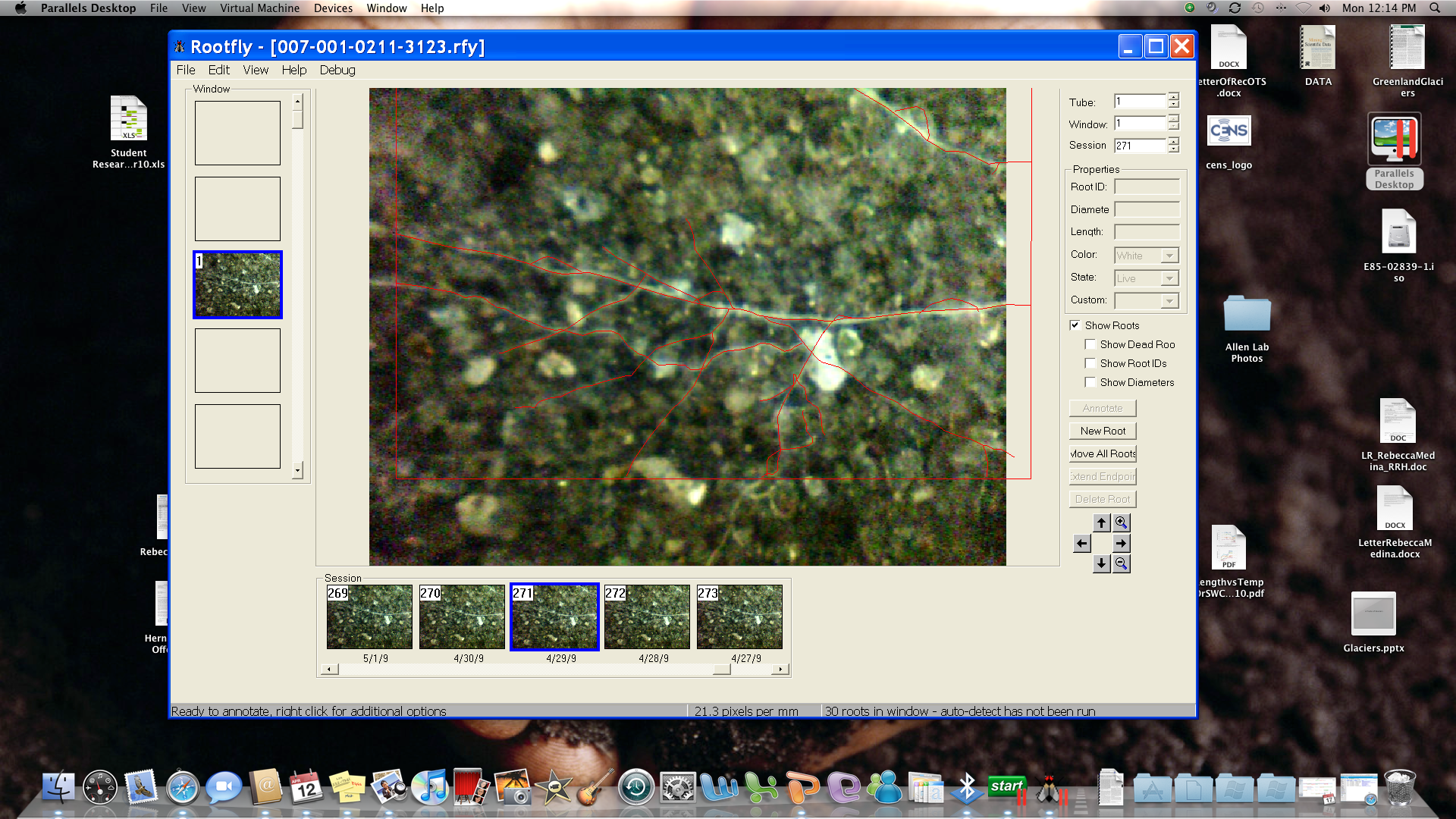


**Fig. S3** A Soil Ecosystem Observatory image/plot (×100) as digitized in Rootfly. Red lines cover the AM fungal hyphae branches.


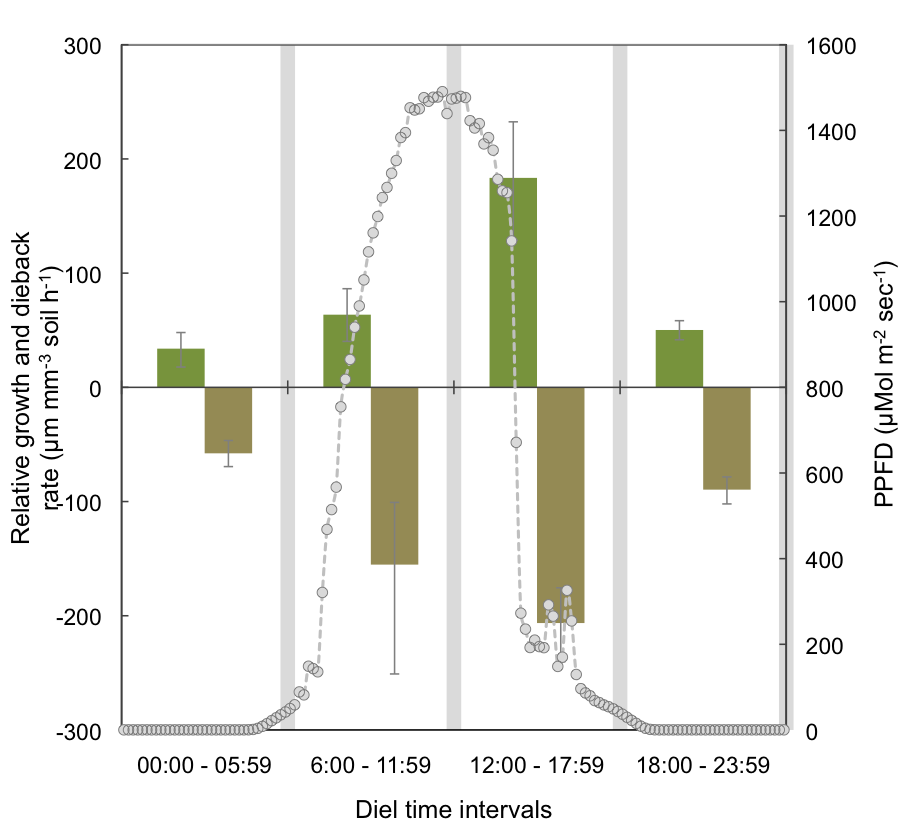

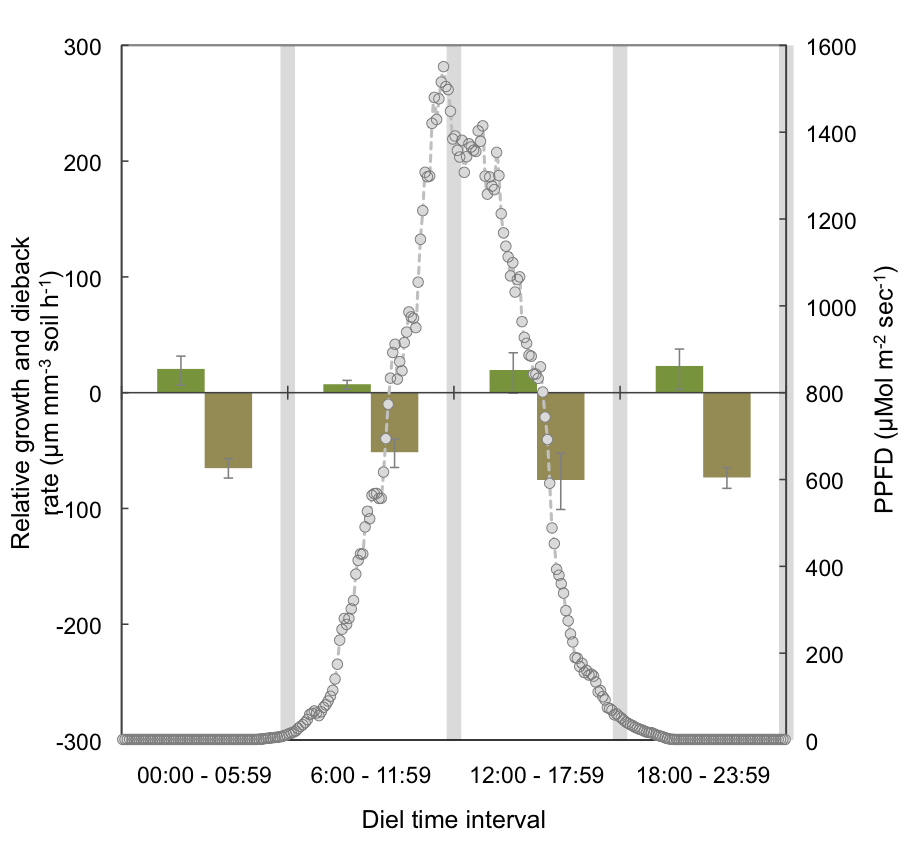


2009

2010

**Fig. S4** Relative diurnal growth (green bars; Kruskal-Wallis chi-squared = 25.505, df = 3, *P* = 1.211e-05 (2009), Kruskal-Wallis chi-squared = 0.4535, df = 3, *P* = 0.929 (2010)) and dieback rates (brown bars; Kruskal-Wallis chi-squared = 77.5848, df = 3, *P* = 2.2e-16 (2009); Kruskal-Wallis chi-squared = 10.741, df = 3, *P* = 0.01321 (2010)) of AM fungal hyphae (μm mm^-3^ soil h^-1^). Photosynthetic photon flux density (PPFD; grey circles) is shown on secondary *y*-axis. Both 2009 and 2010 data quantified from 20 time series analyses of Soil Ecosystem Observatory images (*n* > 1,600) over a 30-d field campaign in May. Error bars are 95% confidence intervals (nonparametric bootstrapping).
